# Editorial Dinner

**Published:** 1879-05-20

**Authors:** 


### *EDITORIAL DINNER.*

The Editors of this Journal had the pleasure of entertaining a number of the Editors of Medical Journals of the United States, at the residence of Dr. Powell, during the Session of the American Medical Association in this city.

About fifteen Medical Journals were represented. There were Parvin, the accomplished scholar and President of the American Medical Association; Dr. Brodie, President of the Editorial Association; Dr. Dunster, the successful teacher in the Michigan University, and Editor; Dr. Reynolds, Kentucky's brilliant optician ; Dr. Bulkely, the American dermatologist; Dr. Cole, the first Vice President of the American Medical Association, from California ; Langdon B. Edwards, the successful young journalist, of Virginia ; Dr. Connor, the able teacher and editor ; Dr. Woodbury, the young and talented representative of the Boston Medical Journal ; Dr. Bell, the able and distinguished sanitarian, of New York ; Dr. Sharpe ; Dr. Jones, of Tennessee ; T.

F. Wood, of N. C.; Dr. Shoemaker, of Philadelphia, and other distinguished adepts and gentlemen of the Medical Profession.

The flow of wit and humor from these gentlemen would, if correctly reported, fill our columns. All were in good spirist, and the toasts and speeches were appropriate, brilliant and sparkling. It was a gathering of talent, culture and scholarship worthy the Medical Profession of any nation. It was an occasion long to be remembered, and one of the most pleasant of our lives. W. T. G.

